# Smoking cessation in pregnant women with mental disorders: a cohort and nested qualitative study

**DOI:** 10.1111/1471-0528.12059

**Published:** 2012-11-21

**Authors:** LM Howard, D Bekele, M Rowe, J Demilew, S Bewley, TM Marteau

**Affiliations:** a PO31 Institute of Psychiatry, King's College LondonLondon, UK; b Women's Health Academic Centre, King's Health Partners, St Thomas' HospitalLondon, UK; c Psychology & Genetics Research Group, Department of Psychology (at Guy's), Institute of Psychiatry, King's College LondonLondon, UK

**Keywords:** Mental disorders, pregnancy, smoking cessation

## Abstract

**Objective:**

To investigate whether 1) pregnant smokers with mental disorders are less likely to accept referrals to smoking cessation services compared with pregnant smokers without disorders; 2) they experience specific barriers to smoking cessation.

**Design:**

Cohort study supplemented by cross-sectional survey and nested qualitative study.

**Setting:**

Three maternity services, London, UK.

**Population:**

Pregnant smokers with and without mental disorders.

**Methods:**

Case notes were examined on a cohort of 400 consecutive pregnant smokers; data were triangulated with routinely collected data on 845 pregnant smokers at two other sites; 27 pregnant smokers were interviewed using qualitative methods.

**Main outcome measures:**

Acceptance of referral to smoking cessation services; perceived barriers to quitting.

**Results:**

Pregnant smokers with a mental disorder recorded by midwives were one-quarter of the cohort (97, 23%), were more likely to accept referral to smoking cessation services (69% versus 56%, adjusted odds ratio 1.70, 95% confidence interval 1.03–2.79), but more likely to still smoke at delivery (69% versus 56%, adjusted odds ratio 2.63, 95% confidence interval 1.41–4.92). Discussion about smoking was documented in 7.7% of subsequent antenatal visits in women with or without mental disorders. Pregnant smokers with diagnosed mental disorders reported that they and health practitioners did not prioritise smoking advice because of concern about adversely impacting mental health.

**Conclusions:**

Pregnant women with mental disorders appear more motivated, yet find it more difficult, to stop smoking. Prioritisation of mental health over smoking may therefore lead to increasing health inequality for this group. Research into effective smoking cessation interventions is required for those with mental disorders.

## Introduction

Smoking is the leading preventable cause of fetal and childhood morbidity and mortality in high-income countries.^1^–^3^ Around 13% of pregnant women in the UK smoke throughout pregnancy,^4^ with similar rates in the USA.^5^ Smoking in women of childbearing age is rising in low-income to middle-income countries, with a predicted prevalence of 20% by 2025,^6^ so it is becoming an important cause of infant death globally.

Women are more likely to stop smoking during pregnancy than at other times^7^; 25–40% have already stopped by the antenatal booking appointment.^8^ Continued smoking in pregnancy is associated with social disadvantage, low income, low education level, mental disorders, domestic violence and low levels of support.^9^,^10^ In the UK, the social patterning of smoking throughout pregnancy is striking, with recent published self-report data varying between 3% and 30% in Westminster and Blackpool respectively.^4^ Similarly, socio-economic disadvantage is strongly associated with antenatal depression,^11^,^12^ so it is unsurprising that almost 50% of pregnant smokers have depression or other common mental disorders.^10^

Smoking cessation programmes in pregnancy reduce the proportion of women who continue to smoke^13^,^14^ and can impact on birth outcomes^15^–^17^ so referral from maternity services is emphasised as an important component of antenatal care in current guidelines.^18^ Although there is evidence that cessation rates can be significantly lower in people with mental disorders,^19^ there has been no research on how mental disorders in pregnancy modify the effectiveness of the current NHS pathway for smoking cessation interventions.

We therefore aimed to investigate whether pregnant women with mental disorders: a) are less likely to accept referrals to smoking cessation services, b) are less likely to stop smoking by delivery, and c) differ in their experiences of smoking, smoking cessation and smoking cessation services compared with pregnant women without mental disorders.

## Methods

### Cohort study

#### Setting

We extracted, as part of an audit of smoking cessation in maternity services locally, routinely collected data (‘booking’, antenatal visits, delivery) from 400 consecutive pregnant women who reported smoking at the time of their first ‘booking’ appointment between 1 January 2010 and 31 May 2011 at an inner city maternity service, a teaching hospital, serving a deprived, multi-ethnic inner city population in southeast London (5000 deliveries per year). Booking and delivery data were available electronically. Detailed clinical data on other antenatal visits were only available from the hand-held paper records. The maternity service operates an opt-out policy for referrals to NHS smoking cessation services as recommended by NICE guidelines (NICE 2010), i.e. all pregnant women who smoke at booking are informed that the midwife will send a referral to smoking cessation services, unless they object. Women who accept are then contacted by telephone by the local smoking cessation services to agree or decline a future appointment. In Southwark, the main borough covered by the service, smoking at delivery is reported as lower than the national average, 5 versus 13.4%.^4^

#### Data from booking visit (electronic records)

Data included sociodemographic information (including self-reported ethnicity; index of deprivation calculated from the woman's postcode using the English Indices of Deprivation 2010^20^ and categorised in deciles of deprivation, with 0–10% being the most deprived and 90–100% the least;) substance abuse history; current mental disorder—defined as either Whooley positive in response to the two ‘Whooley questions’ routinely asked by midwives as recommended by NICE^21^ to identify women with possible depression (see Supplementary material, Appendix S1), or receiving current treatment from general practice or psychiatric services, (a yes to either Whooley question has good specificity (>82%) and reasonable sensitivity (>70%) for screening for depression^22^,^23^ although it has not been validated in pregnancy); past psychiatric history including diagnosis; obstetric history; smoking status (whether or not they smoke); acceptance of referral to smoking cessation services recorded by midwives. Data on contacts by local smoking cessation services and attendances were not routinely available.

#### Data from subsequent antenatal visits (maternity notes)

For each antenatal contact with maternity services, data were collected from the notes on gestational age; health professional seen; documentation relating to smoking, current smoking, quit attempts, attendance at smoking cessation services. Smoking at delivery was recorded electronically.

#### Booking data from other sites

To triangulate findings from the cohort study, and to establish whether the opt-in policy impacted on referral rates differentially for women with mental disorders, supplementary information was gathered from routinely collected electronic data on consecutive pregnant smokers from two other inner London maternity services that use an opt-in policy for referrals to NHS smoking cessation services, i.e. women are not routinely referred but are invited by midwives to consider referral. Data were available for demographic variables, the Whooley questions and current mental health at booking only. We calculated that a sample size of 400 in each group would be sufficient to detect a difference in smoking cessation referral rates from 15% in the pregnant women without mental disorders compared with 7.5% in pregnant women with mental disorders, assuming a significance level *P* = 0.05 (double-sided) at 90% power.

#### Data analysis

Data were analysed using stata v10.1.^24^ The *t* tests and chi-square tests (or Fisher's exact test where cell *n* < 5) were used to compare sociodemographic and clinical characteristics between women with and without a current mental health problem and to compare the characteristics of those who accepted and declined referral to smoking cessation services. Multivariate analyses using logistic regression were used to investigate predictors of acceptance of smoking cessation referral and smoking at delivery, including potential confounders that were found to be related to mental health status in the bivariate analysis at *P* < 0.1. Women who had a fetal death were excluded from the analysis of smoking cessation because smoking at delivery was not recorded; characteristics of excluded women were compared with included women to assess bias. Finally, the relationship between mental health status and documented discussions about smoking at antenatal visits (midwives and obstetricians examined in separate models) was analysed using a multilevel logistic regression model that accounted for the clustering by individual women (using *xtmelogit* in stata), including random intercepts in the model to account for the correlation within individual women.

#### Qualitative interview study

#### Population

Pregnant women with (*n* = 13) and without (*n* = 14) mental disorders who smoked during pregnancy receiving maternity care in southeast London.

#### Procedure

A purposive sample of English-speaking pregnant women who smoked when first pregnant were recruited both from local maternity and perinatal psychiatry services and invited for an interview about smoking cessation to investigate experiences from a range of women who: stopped smoking since becoming pregnant or continued after antenatal booking; attended or declined smoking cessation services; screened Whooley positive or negative for mental disorders at booking; did or did not have a mental disorder needing psychiatric care; and included a range of ages, ethnicities and severities of mental health disorders. Recruitment continued until saturation of themes had been achieved.

#### Measures

Semi-structured qualitative interviews were carried out after piloting. Women were interviewed with the Clinical Interview Schedule—Revised version (CIS-R, a 15–20 minutes structured interview)^25^ to obtain ICD-10 operationalised psychiatric diagnoses. Interviewees were given a £15 gift voucher as compensation for their time. Interviews were audio-taped and transcribed verbatim. For women under psychiatric care, clinical diagnoses were obtained from records with the women's agreement.

#### Analysis

Data were analysed using framework analysis.^26^ Framework analysis involves identifying a thematic framework; charting (involving abstraction and synthesis of key themes); mapping and interpretation of the dataset as a whole. This paper reports only results that illuminate the quantitative cohort study findings.

## Results

### Demographic and background data—cohort study

The sociodemographic and clinical characteristics of participants are provided in Table 1. One hundred and five (26.3%) women were primiparous, and 73 (18.3%) booked after 20 weeks of gestation. Sixty-two (15.5%) said yes to one of the Whooley questions (35 [8.8%] answering ‘yes’ to both questions), and 51 (12.7%) women reported receiving current mental health treatment from a general practice or psychiatric service; so a total of 97 (24.3%) women were classified as having ‘current mental disorders’. Women with current mental disorders were significantly less likely to live with a partner, and were more likely to report substance abuse. There were no other significant differences between the two groups.

**Table 1:** Sociodemographic and clinical characteristics of pregnant smokers with (*n* = 303) and without (*n* = 97) mental health problems

Characteristic (*n* = 400 unless otherwise noted)	No mental disorder*	Mental disorder*	Total	OR	95% CI	Test statistic**	*P*
**Mean age (years), mean (SD)**	27.1 (6.6)	27.5 (6.6)	27.2 (0.3)			−0.05	0.62
**Ethnicity (*n* = 385)**							
White	190 (65.3)	63 (67.0)	253 (65.7)				
Non-white	101 (34.7)	31 (33.0)	132 (34.3)	0.93	0.57–1.51	0.94	0.76
**Index of deprivation (centile) (*n* = 383)**							
0–10%	20 (6.9)	8 (8.6)	28 (7.3)			1.17	
10–20%	112 (38.6)	40 (43.0)	152 (39.7)	0.89	0.36–2.18		0.80
20–30%	82 (27.9)	24 (25.8)	105 (27.4)	0.74	0.29–1.89		0.53
>30%	77 (26.6)	21 (22.6)	98 (25.6)	0.68	0.26–1.77		0.43
**Marital status (*n* = 367)**							
Not married	239 (86.0)	79 (88.8)	318 (86.7)				
Married	39 (14.0)	10 (11.2)	49 (13.4)	0.77	0.37–1.62	0.45	0.50
**Lives with**							
Alone/children	68 (22.4)	35 (36.1)	103 (25.8)				
Husband/partner	153 (50.5)	32 (33.0)	185 (46.3)	0.40	0.23–0.71		0.00
Relatives/friends/other	82 (27.1)	30 (30.9)	112 (28.0)	0.71	0.40–1.27	10.57	0.25
**Substance misuse (*n* = 399)**	39 (12.9)	24 (25.0)	63 (15.8)	2.26	1.27–4.00	8.07	0.00
**Gestation at booking**							
First trimester	74 (57.4)	58 (59.8)	232 (58.0)				
Second/third trimester	129 (42.6)	39 (40.2)	168 (42.0)	0.91	0.57–1.44	0.17	0.68
**Obstetric history**							
Previous perinatal death (*n* = 392)***	4 (1.4)	2 (2.1)	6 (1.5)				0.64
**Obstetric outcome**							
Gestation age (*n* = 334)	39.0 (1.71)	38.3 (3.47)	38.8 (2.26)			2.48	0.01
Birthweight, mean (SD) (*n* = 329)	3096.3 (5,245)	2,955 (711)	3,063 (575)			1.90	0.06
Perinatal death (*n* = 352)	8 (3.0)	6 (6.1)	14 (4.0)	2.46	0.83–7.30	2.79	0.10

* Data are given as *n* (%) unless otherwise stated.

** *t* test or chi-square test unless otherwise stated.

*** Fisher's exact test.

#### Psychiatric history and substance misuse details—cohort study

Diagnosis was only recorded for some women: there were 83 (21%) women who reported a history of diagnosed depression (including 12 previous postnatal depression), five (1.3%) with schizophrenia, three (0.8%) with bipolar disorder, one (0.3%) with an eating disorder and 11 (2.8%) women had a history of self-harm or overdose without a specified diagnosis. Only two women reported that they had had contact with psychiatric services in the previous 12 months, but 43 (10.8%) had seen a psychiatrist previously and 15 (3.8%) had a history of a psychiatric admission. Nineteen (4.8%) women were taking psychotropic medication at booking; 12 (3%) antidepressants, four (1%) antipsychotics, one lithium and one valproate; medication was missing for one woman. Sixty-three (15.8%) women reported some type of substance abuse (one missing data, one type of drug not recorded). Cannabis-only use was reported in almost two-thirds of substance misusers (*n* = 35/399; 8.8%), poly-drug use was also common (*n* = 19; 4.8%) and small numbers of women reported alcohol use >4 units per week (*n* = 4; 1%), cocaine (*n* = 2; 0.5%), and crack use (*n* = 3; 0.8%).

### Outcomes

#### Smoking referral

All women were told a referral to smoking cessation would be made unless they declined—237 (59.3%) women accepted referral to smoking cessation services. Table 2 shows that there were no clinical or sociodemographic differences between women who did and those who did not accept referral, other than women with current mental disorders being significantly more likely to accept a smoking cessation referral (odds ratio [OR] 1.74, 95% confidence interval [95% CI] 1.07–2.84; after adjustment for substance misuse and living alone: adjusted OR [AOR] 1.70, 95% CI 1.03–2.79).

**Table 2:** Characteristics of women accepting (*n* = 237) and declining (*n* = 163) referral

Characteristic	Declined referral*	Accepted referral*	Total	OR	95% CI	Test statistic**	*P*
**Mental health problem**	30 (18.4)	67 (28.3)	97 (24.3)	1.75	1.07–2.84	5.12	0.03
**Age (years), mean (SD)**	27.1 (7.0)	27.1 (6.4)	27.0 (6.6)			−0.09	0.93
**Ethnicity (*n* = 385)**							
White	96 (62.3)	157 (68.0)	253 (65.7)	0.78	0.51–1.20	1.30	0.26
Non-white	58 (37.7)	74 (32.0)	132 (34.3)				
**Index of deprivation (percentile) (*n* = 383)**							
0–10%	13 (8.4)	15 (6.6)	28 (7.3)	1.00	0.98–1.03	0.52	0.67
10–20%	61 (39.6)	91 (39.7)	152 (39.7)				
20–30%	41 (26.6)	64 (28.0)	105 (27.4)				
>30%	39 (25.3)	59 (25.8)	98 (25.6)				
**Marital status (*n* = 367)**							
Not married	122 (85.3)	196 (87.5)	318 (86.7)	0.83	0.45–1.53	0.36	0.55
Married	21 (14.7)	28 (12.5)	49 (13.5)				
Lives with							
Alone/children	40 (24.5)	63 (26.6)	103 (25.8)	0.97	0.74–1.28	0.32	0.85
Husband/partner	78 (47.9)	107 (45.2)	185 (46.3)				
Relatives/friends/other	45 (27.6)	67 (28.3)	112 (28.0)				
Substance misuse (*n* = 399)	24 (14.7)	39 (16.5)	63 (15.8)	1.14	0.66–1.99	0.23	0.63
**Gestation at booking**							
First trimester	86 (52.8)	146 (61.6)	232 (58.0)	0.70	0.46-1.04	3.10	0.08
Second/third trimester	77 (47.2)	91 (38.4)	168 (42.0)				
Obstetric history							
Previous perinatal death (*n* = 392)***	3 (1.9)	3 (1.3)	6 (1.5)			0.23	0.64

* Data are given as *n* (%) unless otherwise stated.

** *t* test or chi-square test unless otherwise stated.

*** Fisher's exact test.

#### Smoking status at delivery

Fourteen women (3.5%) who had a second-trimester or third-trimester fetal death, and one women who had a termination for trisomy 21 were not included in this analysis because of a lack of smoking data at delivery. An additional 48 (12%) women had no delivery data, suggesting that they had moved or delivered elsewhere. There were no significant sociodemographic, clinical or mental health differences in those women with and without smoking at delivery data, other than a significantly higher rate of previous perinatal death in those without delivery data (see Supplementary material, Table S1).

Table 3 shows the sociodemographic and clinical differences in women who reported smoking or not smoking at delivery. White women were significantly less likely to have stopped smoking. Women with mental disorders at booking were significantly more likely to still be smoking than women without disorders (OR 2.60, 95% CI 1.42–4.75; adjusted for ethnicity: AOR 2.63, 95% CI 1.41–4.92).

**Table 3:** Sociodemographic and clinical characteristics of those pregnant smokers who were or were not still smoking at delivery

Characteristic (*n* = 337 unless otherwise noted)	Not smoking at delivery*	Smoking at delivery*	Total	OR	95% CI	Test statistic**	*P*
**Current mental health problems**							
Yes	16 (13.33)	62 (28.6)	78 (23.2)	2.6	1.42–4.75	10.1	0.002
**Age (years), mean (SD)**	27.1 (6.7)	27.1 (6.5)	27.0 (6.6)			0.02	0.98
**Ethnicity (*n* = 325)**							
White	63 (54.8)	146 (69.5)	209 (64.3)	0.53	0.33–0.85	7.03	0.008
Non-white	52 (45.2)	64 (30.5)	116 (35.7)				
**Index of deprivation (*n* = 326)**							
0–10%	9 (7.6)	16 (7.7)	25 (7.7)			1.54	
10–20%	44 (37.3)	89 (42.8)	133 (40.8)	1.13	0.46–2.78		0.78
20–30%	31 (26.3)	55 (26.4)	86 (26.4)	1.0	0.39–2.52		1.00
>30%	34 (28.8)	48 (23.1)	82 (25.2)	0.79	0.31–2.01		0.63
**Marital status (*n* = 311)**							
Not married	95 (84.1)	176 (88.9)	271 (87.1)	0.66	0.34–1.29	1.49	0.22
Married	18 (15.9)	22 (11.1)	40 (12.9)				
**Lives with**							
Alone/children	30 (25.0)	59 (27.2)	89 (26.4)				
Husband/partner	58 (48.36)	94 (43.3)	152 (45.1)	0.82	0.48–1.42	0.78	0.49
Relatives/friends/other	32 (26.7)	64 (29.5)	96 (28.5)	1.01	0.55–1.87		0.96
**Substance misuse (*n* = 336)**	13 (23.6)	42 (76.4)	55 (16.4)	1.96	1.00–3.81	4.00	0.49
**Gestation at booking**							
First trimester	69 (57.5)	119 (54.8)	188 (55.8)				
Second/third trimester	51 (42.5)	98 (45.2)	149 (44.2)	1.11	0.71–1.75	0.22	0.64
**Obstetric history (*n* = 336)**							
Previous perinatal death***	1 (0.83)	2 (0.93)	3 (1.8)			0.008	1.00

* Data are given as *n* (%) unless otherwise stated.

** *t* test or chi-square test unless otherwise stated.

*** Fisher's exact test.

#### Discussions of smoking at antenatal visits

Of the 2393 documented antenatal visits after booking, 324 (13.5%) involved obstetricians (120 consultants, 204 trainees) and 1836 (76.7%) involved midwives. The remaining 233 (9.7%) visits were conducted by other practitioners including general practitioners and substance misuse specialists. Discussions of smoking were recorded for 141 (7.7%) midwife visits, and 25 (7.5%) obstetric visits. There was no difference in recorded discussions of smoking in women with or without current mental disorders by midwives (OR 1.39, 95% CI 0.74–2.6) or obstetricians (OR 0.51, 95% CI 0.16–1.60).

### Cross-sectional routinely collected data

Of the 845 consecutive pregnant smokers (mean age 27.8 years, SD 6.2; 39% non-white) from two inner city maternity services with opt-in smoking cessation policies and routine data available electronically from 2011, 112 (13.3%) had a current diagnosis of a mental disorder recorded, and 111 (13.1%) were Whooley positive. One hundred and seventy-five (21%) women had a current diagnosis or were Whooley positive and were categorised as having a ‘current mental disorder’. There were 453 (53.6%) records of smoking cessation referral offers that were declined by the majority, with only 189 (22.4%) referrals made. Women with a current mental disorder were more likely to accept referral (49%; 51/104) than women without (39.5%; 138/349) (OR 1.47, 95% CI 0.95–2.29; adjusted for ethnicity: AOR 1.62, 95% CI 1.03–2.54).

### Interview study

#### Population

Fifty-three women were invited to participate and 27 consented to be interviewed, of whom 13 were in the second trimester, 13 in the third trimester and one was 4 weeks postnatal. Twenty-one women were recruited from antenatal services (two CIS-R diagnoses for severe depression, three moderate depressive disorder, three mild depressive disorder) and six from perinatal psychiatry services (two bipolar disorder, four recurrent depressive disorder). Participants' mean age was 29 years (SD 1.1, range 17–41), with ethnic variation (13 White, four Black African, six Black Caribbean, four Mixed/Other). The group contained 19 unemployed women, 18 with single marital status, with education status variation (five did not complete compulsory education, ten had obtained GCSEs or NVQs, ten had received a degree or above, two declined to state) and parity variation (nine women had no children, five had one child; six had two to five children); 14 pregnancies were unplanned. Most women reported that they had started smoking as teenagers with friends (three under 13 years old; three after 20 years old). Most reported smoking an average of ten cigarettes/day at the time of interview. Two (one each with bipolar and moderate depressive disorder) had stopped smoking in the second trimester.

#### Perceived barriers to quitting

Themes emerging from the interviews are shown in Table 4. Most were common to both groups of women and included the negative impact of the social and physical environment, and physical addiction. However, women with mental disorders described additional psychological addiction and a different type of relationship with smoking. Examples included: smoking used as a way of losing weight (eating disorder); mainly occurring when manic (bipolar disorder); was the only way to stay well despite the risks (depressive disorder); a quit attempt was feared as it could make the depression worse.

**Table 4:** Barriers to smoking cessation in pregnancy

Theme	Sub-theme	No mental disorder (*n* = 14)	Mental disorder (*n* = 13)
Social environment	Family	√	√
	Partner	√	√
	Social network of peers	√	√
Physical environment	Accessibility of cigarettes	√	√
Addiction	Psychological		√
	Physical	√	√
Influence of mental illness on smoking behaviour	Smoking to help stay thin (eating disorder)		√
	Smoking when acutely ill (manic)		√
Smoking as way of coping with stressful lives (helping emotional wellbeing)		√	√
Services	Judgemental	√	√
	Lack of proactive follow up	√	√
	Lack of continuity of care	√	√
	Prioritisation of mental health over smoking		√

√, evidence of theme or subtheme from interviewees.

There was also some evidence of prioritisation of mental health over smoking by healthcare professionals:

*“*[obstetrician] *was like—don't give up…we don't want you getting anxiety or stressed”*…(woman with recurrent depression)

*“One woman* [referring to mental health social worker]*…said—don't worry about it* [smoking]*”*…(woman with depressive disorder)

## Discussion

### Main findings

Women with current mental disorders were more likely than other pregnant smokers to accept referral to smoking cessation services, whether maternity services used an opt-in or opt-out policy, suggesting that they are just as, if not more, motivated to stop. However, women with mental disorders were more likely to be still smoking at delivery, as has been reported elsewhere.^9^,^10^ The difficulties women with mental disorders have in stopping smoking may be related to limited encouragement and low rates of smoking discussions given by health professionals to all smokers beyond the initial opt-out offer (<8% recorded). Additional barriers may include giving a higher priority to mental health than smoking, found in both women and their maternity and mental health professionals in the qualitative study.

Many of the barriers to smoking cessation, such as the influence of the social and physical environment, smoking as an essential daily coping mechanism, and judgemental unsupportive professionals have been described elsewhere, including in pregnancy.^27^–^29^ However, the lower quit rates and the additional barriers described by pregnant women with mental disorders are consistent with research on people with mental disorders outside pregnancy; this may be because they are more heavily addicted.^30^ In addition, health professionals have not traditionally prioritised smoking cessation in people with mental disorders, possibly because of beliefs that people with mental disorders are less likely to want to stop smoking or that it will worsen the disorder.^31^,^32^ Negative perceptions and attitudes of healthcare professionals have been reported previously as barriers to treating nicotine addiction simultaneously with mental disorders.^33^ In addition, a current focus in the research literature (and on the internet) on the adverse *in utero* impact of ‘stress’ and mental symptoms on the baby^34^–^36^ may mistakenly lead women with mental disorders, and their healthcare professionals, to minimise the significant impact of smoking in pregnancy and prioritise mental health. This has been found in previous studies, which reported that smoking status is less likely to be recorded in medical records of pregnant women with mental illnesses.^37^,^38^

### Strengths and limitations

Strengths of this study include: a substantial cohort of pregnant smokers using routinely collected data on mental health with verification of findings on smoking referral using routinely collected data from two other services; a large diverse sample in the nested qualitative component illuminating the findings; use of diagnostic instruments for mental disorders in the interview study. The main weakness in the study is the lack of data on the use of smoking cessation services after referral and the use of self-reported smoking status at delivery, which is likely to underestimate cigarette use.^39^,^40^ Other limitations include possible under-detection of mental disorders by midwives^41^,^42^ (though misclassification bias would have attenuated any significant differences), limited generalisability from inner London services, possible under-recording of discussions of smoking at antenatal visits and the impact of unmeasured confounders on smoking through pregnancy including the development of mental disorder later in pregnancy and other stressors such as congenital malformations and pregnancy-related maternal complications.

### Implications

No previous study to our knowledge has investigated how mental disorders modify the effectiveness of smoking cessation service delivery during pregnancy. Our findings highlight the need for smoking cessation services to feed back to maternity services whether or not individual women have attended their services and their outcomes, so that maternity professionals can proactively support and encourage women who are finding smoking cessation difficult. Pregnant smokers commonly have mental disorders, and this study suggests a mismatch between their motivation to stop smoking but low success; this may be partly because of concerns that smoking cessation may worsen the mental disorder with an adverse impact on the fetus as seen here in both women and professionals as a reason to continue to smoke. There have been few prospective studies of mental health in pregnancy in women who stop smoking compared with those who do not. However, there is evidence that smoking cessation in pregnancy is associated with decreased depressive symptoms.^43^ Without help for both their mental disorders and smoking, pregnant women with mental disorders will inevitably continue to have worse smoking and obstetric outcomes.^37^,^44^ This study highlights the need to develop more effective smoking cessation interventions for pregnant smokers with mental disorders.

It is plausible that modification of smoking cessation interventions or contemporaneous delivery of mental health interventions alongside a smoking cessation programme are needed to improve smoking, obstetric and mental health outcomes.

Recent systematic reviews have reported that smoking cessation interventions can be just as effective for people with severe mental illnesses and depression (who are being treated for both the mental illness and smoking) compared with the general population,^45^,^46^ though higher rates of abstinence can be achieved in people with depression when involving intensive psychosocial interventions as adjunct to treatment^30^ or cognitive behavioural therapy.^47^ One randomised controlled trial of integrated cognitive behavioural therapy during pregnancy—addressing depression, partner violence, smoking and environmental tobacco smoke exposure in high risk African-American women—reported that mothers in the intervention arm reduced their environmental exposure and were less likely to be smoking postpartum, although there was no impact on smoking rates during pregnancy (possibly related to high spontaneous quit rates in both intervention and control arms).^48^ The intervention also had a significant impact on very preterm birth and very low birthweight.^49^ Although the study needs replication it suggests that integrated interventions may be particularly beneficial, that is addressing the cluster of mental health and other associated problems, such as domestic violence,^50^ alongside smoking cessation.

Population level strategies that impact on environmental smoke exposure and accessibility to cigarettes, and that include education campaigns, are also important. There is evidence from the Sudden Infant Death Syndrome reduction campaign that such campaigns can reduce rates of antenatal smoking in women with severe mental disorders.^51^

## Conclusion

Pregnant women with mental disorders appear more motivated, yet find it more difficult, to stop smoking. Further investigations of smoking cessation interventions for pregnant women with mental disorders are urgently needed. At the maternity service level, there is clearly unrealised potential for health professionals to improve their interactions regarding smoking when seeing pregnant women with mental disorders. Otherwise, the failure to overcome barriers to smoking cessation will continue to compound intergenerational health impacts and inequities for vulnerable and deprived women.

### Disclosure of interest

LMH, JD, SB and TM received a grant from the Foundation for the Study of Infant Death to carry out this research; LMH is also supported by Tommys the Baby Charity and the National Institute for Health Research (NIHR) Mental Health Biomedical Research Centre at South London and Maudsley NHS Foundation Trust and King's College London. All authors have no non-financial interests that may be relevant to the submitted work. There are no other competing interests.

### Contribution to authorship

The authors were involved as follows: LMH for conception, design, data acquisition and supervision, data analysis and interpretation, and drafting the article; DB and MR for design, data acquisition and analysis; JD for conception, data acquisition and interpretation of analysis; SB and TNM for conception, design, data interpretation and drafting of the article; all were involved in revision and final approval.

### Details of ethics approval

The cohort study was part of an audit at King's College Hospital (ref. AP1130-01). The qualitative study and cross-sectional survey using routinely collected data had ethical approval from the Joint South London and Maudsley and the Institute of Psychiatry NHS Research Ethics Committee (the Joint SLAM/IOP REC) (Ref. no 10/H0807/89); interview participants gave informed consent before taking part.

### Funding

This work was funded by the Foundation for the Study of Infant Deaths.

## Supporting Information

**Table S1.** Characteristics of women with smoking data at delivery (*n* = 337) and without (*n* = 63).

**Appendix S1.** Whoolley questions asked by midwives at antenatal booking.
