# Supplementary material for: Smoking cessation in pregnant women with mental disorders: a cohort and nested qualitative study
Source: BJOG. 2012 Nov 21;120(3):362–70. doi: 10.1111/1471-0528.12059 (PMC3638317; doi:10.1111/1471-0528.12059)
Supplement: Supplementary file 1 [file bjo0120-0362-SD1.pdf]

Table S1. Characteristics of women with smoking data at delivery (n=337) and without (n=63)

**Characteristic** (n = 400 unless otherwise noted)	**No Smoking data**	**Smoking data**	**Total**	**OR**	**95% CI**	**Test statistic (t/χ^2)**	**P**
**Mental disorders**	19 (30.2)	78 (23.2)	97 (24.3)	0.70	0.38-1.26	1.42	0.24
**Mean age** (SD)	27.6 (6.9)	27.1 (6.6)	27.2 (6.6)			0.56	0.58
**Ethnicity** (n=385)							
White	44 (73.3)	209 (64.3)	253 (65.7)				
Non-White	16 (26.7)	116 (35.7)	132 (34.3)	1.53	0.82-2.82	1.84	0.18
**Index of deprivation (percentile)** (n=383)							
0-10%	3 (5.3)	25 (7.7)	28 (7.3)				
10-20%	19 (33.3)	133 (40.8)	152 (39.7)	0.98	0.95-1.01	2.08	0.26
20-30%	19 (33.3)	86 (26.4)	105 (27.4)				
>30%	16 (28.1)	82 (25.6)	98 (25.6)				
**Marital Status** (n=367)							
Not married	47 (83.9)	271 (87.1)	318 (86.7)				
Married	9 (16.1)	40 (12.9)	49 (13.3)	0.77	0.35-1.69	0.42	0.52
**Lives with**							
Alone/Children	14 (22.2)	89 (26.4)	103 (25.8)				
Husband/Partner	33 (52.4)	152 (45.1)	185 (46.3)	0.98	0.68-1.41	6.30	0.91
Relatives/Friends/Other	16 (25.4)	96 (28.5)	112 (28.0)				
**Substance misuse**	8 (12.7)	55 (16.4)	63 (15.8)	1.35	0.61-2.98	0.54	0.46
**Gestation at booking**							
First trimester	44 (69.8)	188 (55.8)	232 (58.0)				
Second/third trimester	19 (30.2)	149 (44.2)	168 (42.0)	1.83	1.03-3.28	4.30	0.04
Previous perinatal death	3 (5.4)	3 (0.9)	6 (1.5)			6.35	0.01

*Fisher's exact test
