# Supplementary figures and images for: Smoking cessation in pregnant women with mental disorders: a cohort and nested qualitative study
Source: BJOG. 2012 Nov 21;120(3):362–70. doi: 10.1111/1471-0528.12059 (PMC3638317; doi:10.1111/1471-0528.12059)

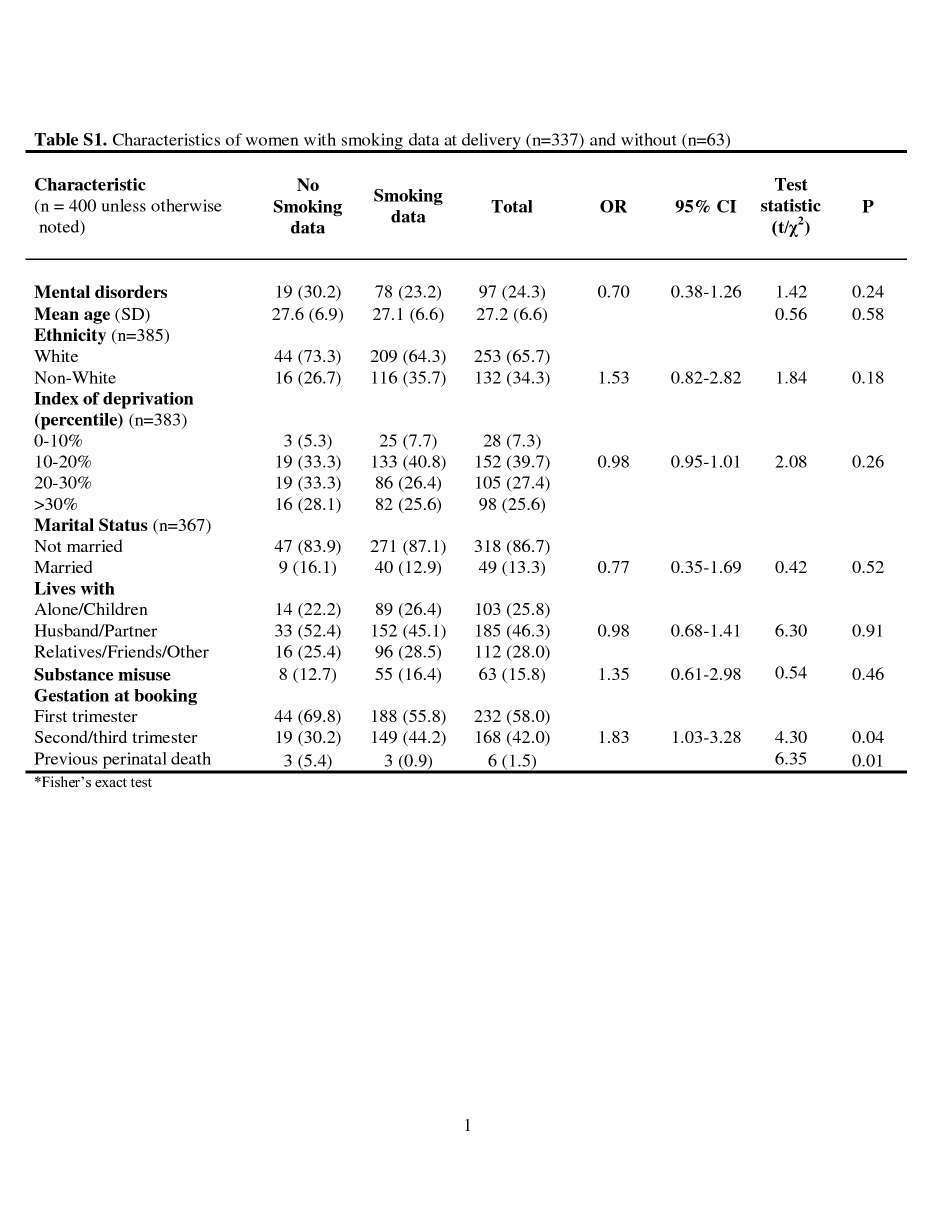

Supplement: Supplementary file 2 [file bjo0120-0362-SD3.png]

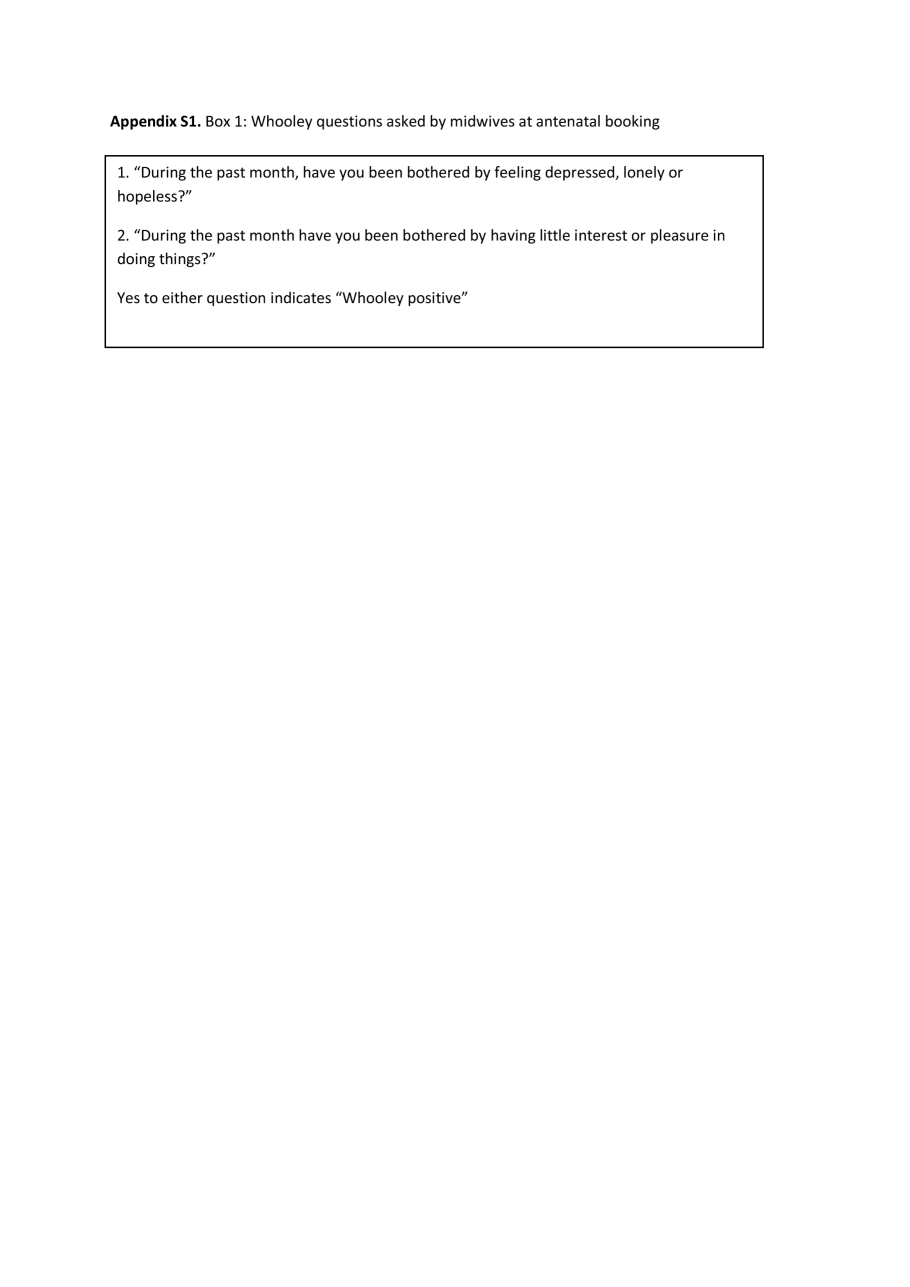

Supplement: Supplementary file 5 [file bjo0120-0362-SD4.png]
