# Supplementary material for: Smoking cessation in pregnant women with mental disorders: a cohort and nested qualitative study
Source: BJOG. 2012 Nov 21;120(3):362–70. doi: 10.1111/1471-0528.12059 (PMC3638317; doi:10.1111/1471-0528.12059)
Supplement: Supplementary file 4 [file bjo0120-0362-SD2.pdf]

Appendix S1. Box 1: Whooley questions asked by midwives at antenatal booking

1. “During the past month, have you been bothered by feeling depressed, lonely or hopeless?”

2. “During the past month have you been bothered by having little interest or pleasure in doing things?”

Yes to either question indicates “Whooley positive”
